# Fabrication of Polycaprolactone-Based Polyurethanes with Enhanced Thermal Stability

**DOI:** 10.3390/polym16131812

**Published:** 2024-06-26

**Authors:** Jasna V. Džunuzović, Ivan S. Stefanović, Enis S. Džunuzović, Tijana S. Kovač, Dušan P. Malenov, Andrea Basagni, Carla Marega

**Affiliations:** 1 Center of Excellence in Environmental Chemistry and Engineering, Institute of Chemistry, Technology and Metallurgy, University of Belgrade, Njegoševa 12, 11000 Belgrade, Serbia; 2 Department of Chemistry, Institute of Chemistry, Technology and Metallurgy, University of Belgrade, Njegoševa 12, 11000 Belgrade, Serbia; istefanovic@chem.bg.ac.rs; 3 Faculty of Technology and Metallurgy, University of Belgrade, Karnegijeva 4, 11100 Belgrade, Serbia; edzunuzovic@tmf.bg.ac.rs; 4 Innovation Center, Faculty of Technology and Metallurgy, University of Belgrade, Karnegijeva 4, 11100 Belgrade, Serbia; tradoman@tmf.bg.ac.rs; 5 University of Belgrade—Faculty of Chemistry, Studentski trg 12–16, 11000 Belgrade, Serbia; malenov@chem.bg.ac.rs; 6 Department of Chemical Sciences, University of Padova, Via Marzolo 1, 35131 Padova, Italy; andrea.basagni@unipd.it (A.B.); carla.marega@unipd.it (C.M.)

**Keywords:** waterborne polyurethane, polycaprolactone, TiO_2_ nanoparticles, thermal stability

## Abstract

The benefit of being acquainted with thermal properties, especially the thermal stability of polyurethanes (PU), and simplified methods for their improvement is manifold. Considering this, the effect of embedding different amounts of unmodified and surface-modified TiO_2_ nanoparticles (NPs) within PU, based on polycaprolactone (PCL) and Boltorn^®^ aliphatic hyperbranched polyester, on PU properties was investigated. Results obtained via scanning electron microscopy, swelling measurements, mechanical tests and thermogravimetric analysis revealed that TiO_2_ NPs can be primarily applied to improve the thermal performance of PU. Through surface modification of TiO_2_ NPs with an amphiphilic gallic acid ester containing a C12 long alkyl chain (lauryl gallate), the impact on thermal stability of PU was greater due to the better dispersion of modified TiO_2_ NPs in the PU matrix compared to the unmodified ones. Also, the distinct shape of DTG peaks of the composite prepared using modified TiO_2_ NPs indicates that applied nano-filler is mostly embedded in soft segments of PU, leading to the delay in thermal degradation of PCL, simultaneously improving the overall thermal stability of PU. In order to further explore the thermal degradation process of the prepared composites and prove the dominant role of incorporated TiO_2_ NPs in the course of thermal stability of PU, various iso-conversional model-free methods were applied. The evaluated apparent activation energy of the thermal degradation reaction at different conversions clearly confirmed the positive impact of TiO_2_ NPs on the thermal stability and aging resistance of PU.

## 1. Introduction

The simplicity of polyurethane (PU) synthesis, the wide range of available reactants and the diversity of PU forms and properties have led, without any doubt, to the significant versatility of applications of PUs [1,2,3]. Materials based on PUs, with excellent mechanical properties, flexibility and elasticity, noticeable solvent and wear resistance and low thermal conductivity, are in most applications (medical devices, packaging, building insulations, coatings, etc.) irreplaceable cutting-edge materials. The credit for the high adaptability of thermal, mechanical, biological and other important properties of PUs goes to the appreciable selection of raw materials and additives, and the ability to easily manipulate and control the formation and extent of microphase separation, present as a result of the thermodynamic immiscibility between hard (isocyanates and chain extenders) and soft segments (polyols). The appearance and extent of microphase-separated domains in PUs are governed by the chemistry and structure of the components, synthetic procedure, polyol molecular weight, proportion between isocyanate, chain extender and polyol, type and portion of the applied additives, etc. [4].

From the aspect of processing and commercial application, one of the most important properties of polymers is thermal stability. The knowledge of the number and type of thermal decomposition processes of polymers is especially important for designing novel materials with desired performances at high temperatures. Thermal degradation of PUs is a complex, multi-step process, usually accompanied by various chemical and physical phenomena [5,6,7,8]. In the simplest case, thermal decomposition of segmented PUs can be considered a two-step process. The first step (>200 °C) is ascribed to the thermal degradation of the weakest urethane linkages (hard segments, HS), while during the second step, thermal decomposition of soft segments (SS) occurs. The initial temperature of the first (faster) step depends on the structure of the applied isocyanate. On the other hand, the initial temperature of the much slower second step depends on the SS structure. Generally, the thermal stability of PUs is poor and needs to be improved in order to increase the diversity of PU applications.

One of the frequently applied methods to control and enhance the thermal stability and other multifaceted properties of PUs is based on the incorporation of different nanoparticles (NPs) with adequate chemical natures, sizes, shapes, morphologies, physicochemical properties and the capability to modify their surface in order to favor and encourage desired interactions with PU chains [9,10,11,12,13,14,15,16,17,18,19]. Among different NPs, titanium dioxide (TiO_2_) NPs turned out to be particularly interesting as reinforcement agent for polymers due to their versatility, large effective surface area, great chemical and physical stability, non-toxicity, biocompatibility, high photoactivity and thermal conductivity, good mechanical, electrochemical, photocatalytic, antimicrobial and UV-protecting properties, and low cost. Although TiO_2_, as a polymorphic compound, exists in nature in three main mineral forms (anatase, rutile and brookite), the majority of the published papers related to TiO_2_-based polymer composites deal with the anatase and rutile forms [20,21,22,23,24]. The synergistic effect of TiO_2_ NPs and PUs has been thoroughly explored and discussed in the literature. It has been shown that the incorporation of rutile TiO_2_ NPs in PUs improves their UV resistance, while the anatase form of TiO_2_ NPs is mainly used as a photocatalyst in PU/TiO_2_ composites [25]. Alam et al. revealed that PU/TiO_2_ nanocomposites (NCs) displayed improved thermal, corrosion and mechanical properties compared to pure PU coatings based on sunflower oil [15]. Also, the thermal stability of PU based on polycarbonate was notably enhanced after the inclusion of TiO_2_ NPs [26]. The same was detected for PU/TiO_2_ composites prepared from PCL diol by Silva et al. [27]. Furthermore, Yang et al. studied the properties of PU/TiO_2_ membranes and they discovered, amongst others, that the thermal stability of fabricated membranes increases with an increase in TiO_2_ NP content [28]. Likewise, the onset temperature of the thermal decomposition of PU shifted from 360 to 380 °C after the incorporation of NH_2_-modified TiO_2_ NPs [29].

Herein, PU/TiO_2_ composites were fabricated using an environmentally safe PU network prepared from polycaprolactone (PCL) and different amounts of unmodified TiO_2_ NPs and TiO_2_ NPs surface modified with lauryl gallate. With the aim to reveal the impact of unmodified and surface-modified TiO_2_ NPs and their content on the morphology, mechanical properties and thermal stability of PUs, the performances of PU/TiO_2_ composites were examined via scanning electron microscopy, swelling measurements, mechanical tests and thermogravimetric analysis, and compared with the reference, pure PU. To further investigate the thermal degradation process of prepared samples, various iso-conversional model-free methods were applied. By monitoring thermal degradation measurements in a nitrogen atmosphere at different heating rates, it was possible to estimate the apparent activation energy of the thermal degradation reaction in the examined samples at different conversions. To the best of our knowledge, this kind of study on PU/TiO_2_ composites fabricated from PCL and TiO_2_ NPs surface modified with lauryl gallate is lacking in the literature. 

## 2. Materials and Methods

### 2.1. Materials

Polycaprolactone diol (PCL, *M*_n_ ≈ 2000 g/mol), supplied by Sigma-Aldrich (Darmstadt, Germany), was dried at 80 °C for 12 h before use. Boltorn^®^ hyperbranched polyester of the second pseudo generation, with an average number of 12 end –OH groups [30], was purchased from Perstorp Specialty Chemicals AB (Perstorp, Sweden) and dried in a vacuum oven for 48 h at 50 °C. *N*,*N*-Dimethylacetamide (DMAc) was obtained from Sigma-Aldrich and distilled under reduced pressure over calcium hydride before being used to prepare a catalyst solution. Tetrahydrofuran (THF) was supplied by J. T. Baker (Phillipsburg, NJ, USA) and kept over molecular sieves (0.4 nm) after being distilled over lithium aluminum hydride. Commercial TiO_2_ NPs (P25, size 25 nm) were purchased from Degussa, while isophorone diisocyanate (IPDI), toluene, stannous octoate, lauryl gallate (LG) and acetonitrile (AN) were purchased from Sigma-Aldrich and used as received.

### 2.2. Surface Modification of TiO_2_ Nanoparticles 

The surface of commercial TiO_2_ NPs was modified with lauryl gallate by adding 2 g of TiO_2_ into a 100 cm^3^ solution of LG in AN (0.01 mol/dm^3^). After keeping the mixture in an ultrasonic bath for 10 min and leaving overnight, surface-modified TiO_2_ NPs (TiO_2_–LG) were precipitated in a centrifuge, washed with AN twice and dried at 40 °C in a vacuum oven until the weight was constant. 

### 2.3. Synthesis of Pure Polyurethane and Composites 

Pure PU with a PCL (soft segment) content of 30 wt.% was synthesized via a two-step polymerization reaction in solution, as described in [8]. The stoichiometric ratio between –NCO and –OH groups was 1.1. During the first step of the polymerization reaction, the solution of PCL (2.80 g, 1.4 mmol) in 11.8 mL THF was heated to 80 °C in a four-necked flask using an oil bath. When 80 °C was reached, 0.68 g of IPDI (3.06 mmol) and 0.14 mL of a catalyst solution in DMAc (0.15 mol% based on PCL) were poured into the mixture. After 3 h of continuous stirring at 80 °C, an NCO-terminated prepolymer was obtained. The content of −NCO groups was controlled by a dibutylamine back-titration method [31]. During the second step of the polymerization reaction, 2.95 g of hyperbranched polyester (2.20 mmol) was dissolved in 10.6 mL of DMAc and, together with 2.89 g of IPDI (13.00 mmol), was added dropwise and finally stirred for an additional 15 min. After pouring the reaction mixture into Teflon dishes, curing of the prepared PU was performed by heating it in an oven at 60 °C for 24 h and then at 80 °C for 3 h. The final curing of pure PU was done in a vacuum oven at 50 °C for 24 h.

Polyurethane composites with unmodified and surface-modified TiO_2_ NPs were prepared in the same manner as pure PU, by incorporating different quantities of NPs (0.5, 1.0 or 2.0 wt.% based on the total weight of PCL, hyperbranched polyester and IPDI) during the first step of the polymerization reaction. Before inserting NPs into the reaction flask, mixtures of adequate amounts of TiO_2_ NPs and THF were stirred in an ultrasonic bath for 20 min. Six composites with different content of TiO_2_ NPs were labeled as PUC0.5, PUC1.0 and PUC2.0 (prepared with 0.5, 1.0 and 2.0 wt.% of unmodified TiO_2_ NPs, respectively) and PUC_M_0.5, PUC_M_1.0 and PUC_M_2.0 (prepared with 0.5, 1.0 and 2.0 wt.% of TiO_2_–LG NPs, respectively).

### 2.4. Characterization

FTIR spectra were recorded using an FT-IR Nicolet SUMMIT (Thermo Scientific, Waltham, MA, USA) spectrometer operating in ATR mode, in the range of 400–4000 cm^−1^, with 64 scans and a spectral resolution of 2 cm^−1^.

In order to inspect swelling behavior, square specimens of pure PU and composites (10.0 mm × 10.0 mm × 1.0 mm ± 0.2 mm) were immersed in THF at room temperature, and their weight was periodically measured. From the data obtained by swelling measurements, the values of swelling degree (*q*), gel content (gel%), volume fraction of the crosslinked polymer in the swollen specimen (*V*), crosslinking density (*ν*) and average molecular weight of the polymer chain between crosslinks (*M*_c_) of all samples were evaluated according to the equations and procedures given in the literature [32,33]. All calculated parameters represent the average of three measurements.

To calculate the density of the prepared samples at room temperature, distilled water was used as the medium and a pycnometer was applied. For each sample, a minimum of three measurements were carried out. 

Scanning electron microscopy (XL30 SEM, Philips, Eindhoven, The Netherlands), equipped with a field emission gun source and Gemini column (Zeiss Supra 35 VP, Oberkochen, Germany), was utilized to examine the cross-sectional morphology of fracture surfaces of the prepared samples. Before measurements were taken in a high-vacuum evaporator, the samples were gold-coated.

The mechanical properties of pure PU and the composites (dimensions 35 mm × 10 mm × 0.5 mm ± 0.2 mm), at room temperature, were examined via an Instron Model 3300 mechanical tester (Instron, Norwood, MA, USA), using stress-strain measurements and a strain rate of 2 mm/min. Three different estimations were conducted for each sample. 

Thermal stability and evaluation of the apparent activation energy of the thermal degradation reaction of the samples at different conversions were accessed from the data obtained by thermogravimetric analysis (TGA), performed on a TA Instrument SDT 2960 simultaneous TG/DSC system. Measurements were conducted under an inert atmosphere (N_2_ gas), in the temperature range from room temperature to 700 °C, using four different heating rates (5, 10, 15 and 20 °C/min). 

## 3. Results and Discussion

A pure PU network with 30 wt.% PCL content and six polyurethane composites with different portions of unmodified or surface-modified commercial TiO_2_ NP were prepared using a two-step polymerization reaction in solution. With each type of TiO_2_ NPs (unmodified and surface-modified), three samples with 0.5, 1.0 and 2.0 wt.% of TiO_2_ NPs were prepared. Surface modification of TiO_2_ NPs was done in order to facilitate the interfacial interaction of hydrophobic PU with hydrophilic TiO_2_ NPs and to enable their adequate insertion in the polymer matrix. For this purpose, an amphiphilic gallic acid ester with a C12 long alkyl chain (lauryl gallate) was utilized. The long aliphatic chain in LG represents the hydrophobic fragment, while –OH groups attached to the benzene ring are regarded as the hydrophilic part of the gallic acid ester. The surface modification of commercial TiO_2_ NPs was accomplished by forming a bridging complex between adjacent –OH groups attached to the benzene ring in LG and the surface Ti atoms of TiO_2_ NPs [34,35,36]. Moreover, to assist the homogenous distribution of applied nano-fillers in the polymer, a mixture of TiO_2_ NPs and THF was stirred in an ultrasonic bath before the polymerization reaction. 

### 3.1. FTIR Spectroscopy

**Figure 1:**
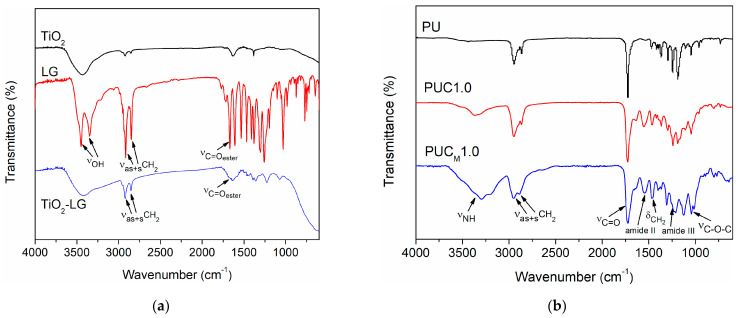
FTIR spectra of (**a**) lauryl gallate (LG), unmodified (TiO_2_) and surface-modified (TiO_2_-LG) NPs and (**b**) pure PU and composites with 1.0 wt.% of nano-filler.

The surface modification of TiO_2_ NPs through the adjacent –OH groups attached to the benzene ring was confirmed with FTIR spectroscopy (Figure 1a). It was observed that the FTIR spectra of LG displayed peaks related to the –OH stretching at 3450 and 3348 cm^−1^, while these peaks were not visible in the spectra of TiO_2_–LG NPs. On the other hand, peaks at 2916 and 2847 cm^−1^, attributed to the stretching vibration of C–H bonds in the lauryl group, as well as a band at 1668 cm^−1^, corresponding to the C=O stretching of the ester group, were present in both spectra [34,35,36]. Representative FTIR spectra of pure PU and both groups of composites, prepared with 1.0 wt.% of unmodified or modified TiO_2_ NP, are shown in Figure 1b. The absence of a band at 2270 cm^−1^ verified that all –NCO groups were fully transformed into urethane linkages. Additionally, other characteristic peaks could be observed at around 3340 cm^−1^ (stretching vibrations of H-bonded urethane N-H groups), at 2950 and 2865 cm^−1^ (asymmetric and symmetric C-H stretching, respectively), at 1465 cm^−1^ (deformation vibrations of CH_2_ groups), at 1548 and 1241 cm^−1^ (amide II and amide III vibrations, respectively), at 1070 and 1040 cm^−1^ (asymmetric and symmetric stretching of C–O–C groups, respectively) and between 1640 and 1730 cm^−1^ (stretching vibrations of C=O groups) [8]. 

**Figure 2:**
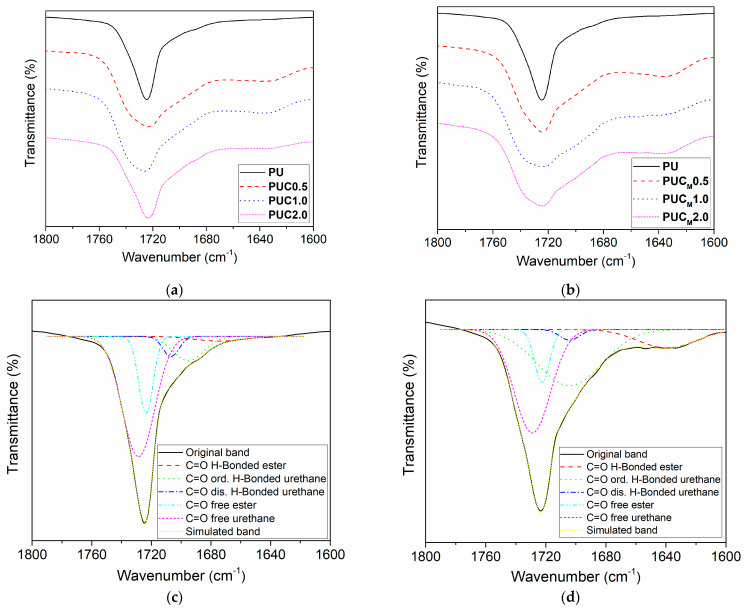
Enlarged C=O stretching region of pure PU and composites prepared with (**a**) unmodified and (**b**) modified TiO_2_ NPs, and C=O stretching region of (**c**) pure PU and (**d**) composite PUC2.0 after applying Gaussian deconvolution.

The influence of TiO_2_ NPs on the hydrogen bond formation and microphase separation in the PU network was studied by detailed examination of the carbonyl region using the Gaussian deconvolution approach (Figure 2, Table 1). Five peaks could be observed in the C=O region, including free urethane C=O groups at 1729 cm^−1^, hydrogen-bonded urethane C=O groups in disordered domains at 1705 cm^−1^, hydrogen-bonded urethane C=O groups in ordered domains at 1695 cm^−1^, free ester C=O groups at 1722 cm^−1^ and hydrogen-bonded ester C=O groups at 1640 cm^−1^. The results listed in Table 1 indicate that microphase separation is greater in the composites than in pure PU and it is more pronounced in the composites prepared with modified TiO_2_ NPs. The latter conclusion was drawn by comparing the ratio between the area originating from the hydrogen-bonded urethane C=O groups in ordered domains and the sum of the area belonging to the hydrogen-bonded urethane C=O groups in disordered domains and free urethane C=O groups.

### 3.2. Swelling Measurements

**Figure 3:**
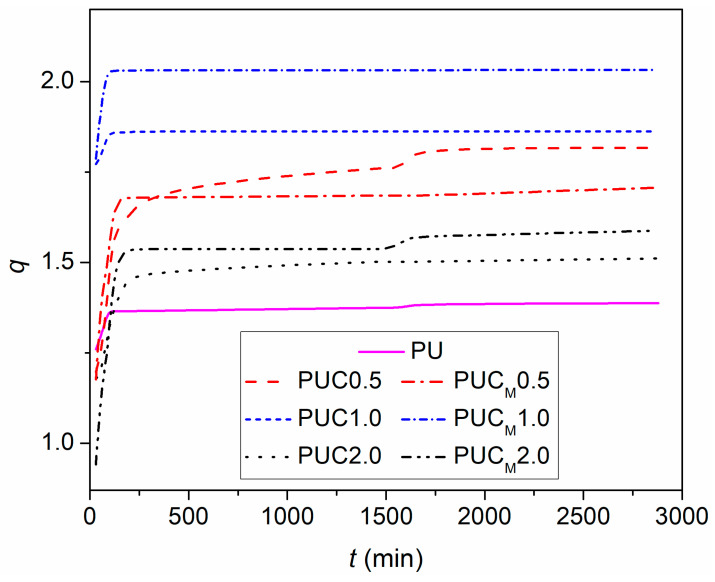
Swelling degree (*q*) of prepared samples in THF vs. time (*t*).

To identify the effect of NP incorporation in the PU network on its swelling ability, swelling measurements of all samples in THF were conducted. It has been shown that PU networks based on PCL and hyperbranched polyester reach an equilibrium swelling degree in THF after 48 h [8,33]. The dependences of the swelling degree on time for prepared samples are compared in Figure 3. It appears that the incorporation of TiO_2_ NPs increases the swelling degree of the PU network, regardless of whether they are modified or not, or the content at which they were added. With increasing the content of the TiO_2_ NPs from 0.5 to 1.0 wt.%, the swelling degree of both sets of samples increases as well. However, further increasing the nano-filler content reduced the swelling ability of the PU matrix. Obviously, in the presence of applied TiO_2_ NPs, the movements of polymer chains in the PU network are not as restricted as in pure PU, indicating the absence of covalent bonding between the nano-fillers and the polymer matrix. The easier and facilitated movement of chains in the polymer network filled with NPs is probably caused by the hindering effect of NPs, whose presence interferes with the formation of crosslinks. Hydroxyl groups at the surface of unmodified commercial TiO_2_ NPs can interfere and compete with the reaction of –OH groups of PCL and hyperbranched polyester during the polymerization and crosslinking processes [27]. Furthermore, in the case of modified TiO_2_ NPs, the presence of long alkyl chains in LG can impede the access of crosslinking agents to the possible crosslinking points. Therefore, when the investigated composite specimens were immersed in a medium applied for swelling, their diffusion through the PU network was easier than in the case of pure PU. The decline in the swelling degree of the samples prepared with 2 wt.% of nano-filler (but still above *q* of pure PU) reveals the existence of another opposite effect of NPs. Namely, the presence of a larger amount of TiO_2_ NPs, especially those modified with LG, hinders not only the diffusion of THF through the PU network, but also the chain movements, probably due to the presence of agglomerates. Their appearance represents an additional obstacle in the THF pathway through the PU network and intrudes further *q* increase. 

To check these assumptions, values of the crosslinking density (*ν*), average molecular weight of polymer chains between crosslinking points (*M*_c_) and gel content for all samples were evaluated using the results gained by the swelling measurements in THF (Table 2). As expected, the crosslinking density of all samples was of the same order of magnitude, but pure PU had the highest value of *ν*. Consequently, the samples with a lower *ν* had lower gel content and a higher *M*_c_. Another interesting observation was that with an increase in NP content, a small increase in the crosslinking density occurred as well. The slight increase in *ν* with an increasing amount of TiO_2_ NPs from 0.5 to 1.0 wt.% can be ascribed to experimental error, while the obtained values for composites with 2 wt.% of NPs clearly indicate a lower swelling ability. In the structure of the PU network, two types of crosslinks exist: chemical and physical. Chemical crosslinks are created through chemical reactions involving a crosslinking agent, while the physical network in PU is set up as a result of hydrogen bonding between urethane groups of adjacent chains. However, the crosslinking density evaluated from swelling measurements takes into account only the presence of the chemical network in the investigated polymer, and that may be the reason for the obtained trend of *ν* with increasing content of NPs as well [33].

### 3.3. SEM Analysis

**Figure 4:**
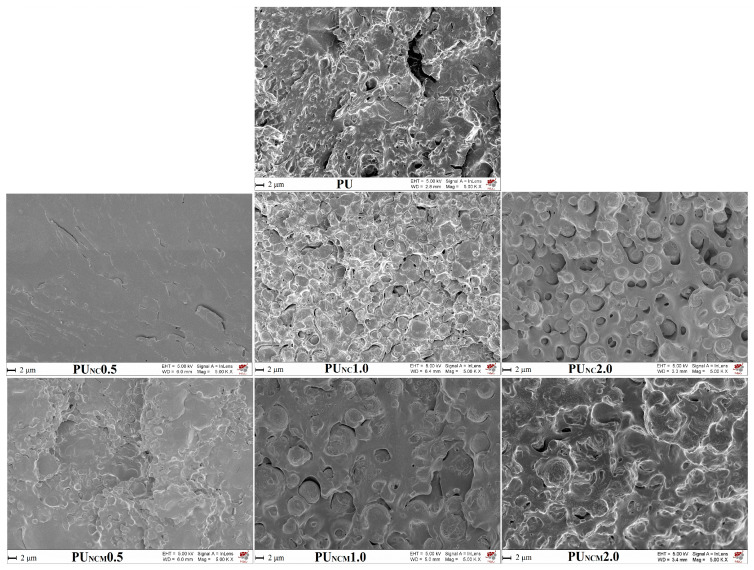
SEM micrographs of cross-sectional surfaces of prepared samples.

To visualize the morphology of the fracture surfaces of the prepared samples, SEM was applied, and the derived images are shown in Figure 4. The SEM image of pure PU shows the existence of rough and irregular textures on the cross-sectional surface [8]. An irregular surface texture can also be seen in the SEM images of composites with 0.5 wt.% of unmodified and modified TiO_2_ NPs, but it is less pronounced (especially in the SEM image of PUC0.5). With increasing the content of NPs, the morphology of the composites’ fracture surfaces changed. Namely, the dispersion of nanoparticles in PU could deteriorate if excessive content of NPs was applied, leading to the formation of agglomerates and the emergence of inconsistent properties. It could be observed that a rise in TiO_2_ NP content induced the appearance of a rougher fracture surface, sheltered with spherical forms of different sizes, probably as a result of NP agglomeration. When comparing SEM images of PUC and PUC_M_ samples, it is obvious that the inclusion of surface-modified TiO_2_ NPs led to the appearance of microspheres, which are less visible and better dispersed in the polymer matrix due to the presence of a hydrocarbon chain attached to the surface of the TiO_2_ NPs. 

### 3.4. Mechanical Properties

**Figure 5:**
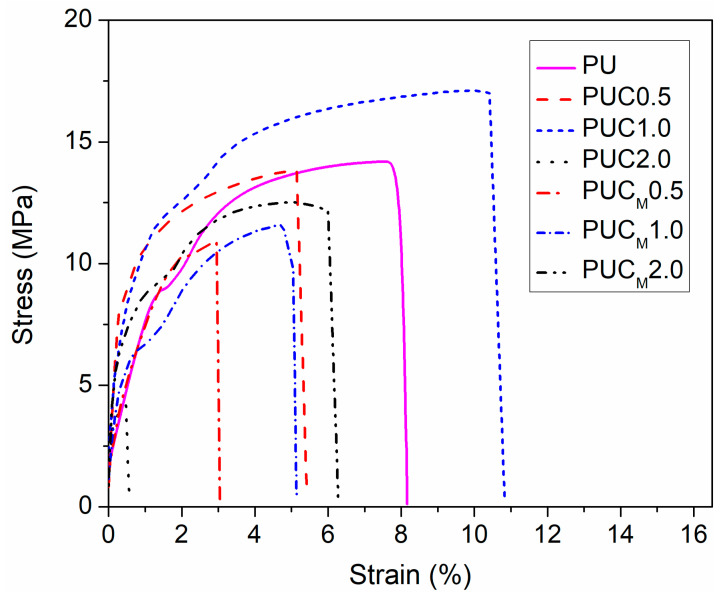
Tensile stress-strain dependence of prepared samples.

The mechanical properties of composites prepared through the incorporation of NPs in the polymer matrix depend on the strength of the polymer/NPs interfacial region, which is strongly affected by the type of bonding between matrix and filler (either covalent or van der Waals) and quality of the NP distribution in the polymer (homogenous or heterogeneous). Higher strength of the polymer/NP interfacial region/bonding and homogenous distribution of NPs provide better and more even transfer of interfacial stress, consequently increasing the tensile strength of such composites. On the other hand, the absence of covalent bonding and/or presence of agglomerates, especially when a higher content of NPs is used, induces concentration of stress and decreases tensile strength when force is applied on such samples. Results presented in Figure 5 and Table 3 show that from the perspective of mechanical properties, the incorporation of unmodified or surface-modified TiO_2_ NPs in the PU network based on PCL may be disadvantageous. Only the sample with 1.0 wt.% of TiO_2_ NP loading showed some increase in tensile strength. However, further increasing the content of unmodified TiO_2_ NPs in PU significantly reduced the tensile strength of the PU network, which again indicates that TiO_2_ NPs are gathered in agglomerates in PUC2.0, forming in this manner new, extra stress centers. These findings are in agreement with the SEM results. A similar effect of TiO_2_ NPs on the mechanical properties of PUs can be found in the literature [27,37]. The inclusion of surface-modified TiO_2_ NPs in PU aggravates the mechanical properties of PU as well, but variations in their content show no spectacular influence on the tensile strength, elongation at break and Young’s modulus values.

### 3.5. Thermal Stability 

**Figure 6:**
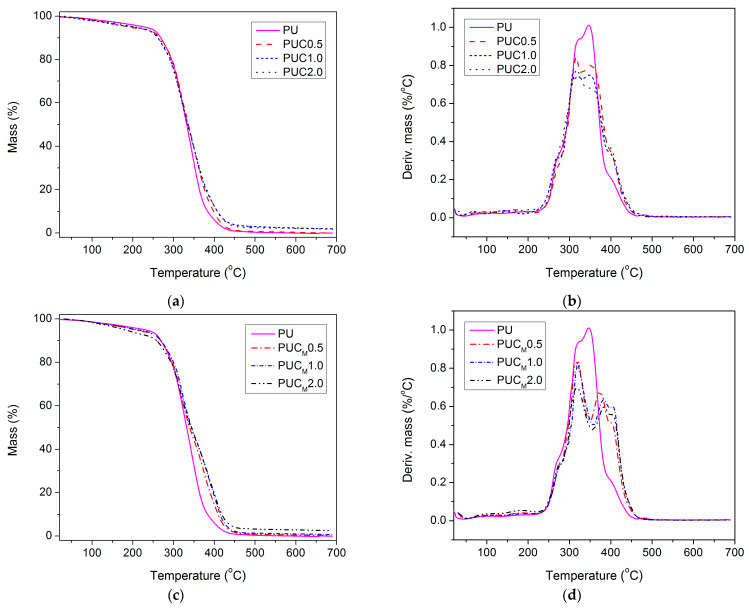
(**a**) TG and (**b**) DTG curves of pure PU and PUC samples, (**c**) TG and (**d**) DTG curves of pure PU and PUC_M_ samples, determined at a heating rate of 10 °C/min.

The application lifetime of polymer materials greatly depends on their thermal performances and behavior at elevated temperatures. If the service temperature is too high for the applied polymer material, its thermal degradation is an unavoidable process. The incorporation of NPs in a polymer matrix can shift its decomposition temperature to higher values and expand the temperature range of material use [9,10,11,12,13,14,15,16,17,18,19,25,26,27,28,29]. In order to examine the thermal behavior of pure PU and the prepared composites, TGA was completed, and the data gained at a heating rate of 10 °C/min are shown in Figure 6 and listed in Table 4. All TG curves had the same shape and the initial thermal degradation temperature (at 10% weight loss) was similar for all examined samples (between 262 and 269 °C), except for PUC_M_2.0, for which a certain deviation was observed. This can be elucidated in consonance with Figure 6d. Namely, the small but broad peak below 200 °C in all DTG curves, attributed to the evaporation of residual solvent (and other evaporable constituents), is the most pronounced in PUC_M_2.0. This further indicates the presence of a higher amount of solvent trapped during the synthesis of this sample than in others [8]. 

Based on the TG and DTG curves, it is evident that the thermal degradation of pure PU and the prepared composites is a two-step process. For the appearance of the first DTG peak around 320 °C, the decomposition of weak urethane linkages present in HS is responsible [5,6,7,8]. There is a slight shift in the position of this DTG peak to lower temperatures for the composites, and a noticeable decrease in the maximum thermal decomposition rate compared to the DTG results of pure PU. This can be explained by taking into account the lower crosslinking density of the composites. Also, the presence of a higher content of low molecular weight evaporable compounds in the composites could be an additional reason for the lower position of the first DTG peak [38,39]. On the left side of the first DTG peak, a shoulder of lower intensity could be observed in all examined samples (*T*_sh1_, Table 4), which could also be ascribed to the degradation of urethane bonds. 

By comparing the TG and DTG curves of pure PU and the prepared composites, the positive influence of TiO_2_ NPs on thermal stability is particularly visible at higher temperatures, i.e., at temperatures at which thermal decomposition of PCL soft segments occurs (position of the second DTG peak, *T*_max2_). Besides, the shift of the second DTG maximum to the higher temperatures was more pronounced for composites prepared using TiO_2_ NPs surface modified with lauryl gallate. Also, the *T*_max2_ of PUC_M_ samples was slightly displaced to the higher values with an increase in the content of TiO_2_–LG NPs, while the *T*_max2_ of PUC showed no change with an increase in the content of unmodified TiO_2_ NPs. The aliphatic part of lauryl gallate was obviously long enough to secure better dispersion of TiO_2_–LG NPs in PU, simultaneously leading to the improvement of the PU thermal properties. Another observation could also be made. Namely, for pure PU and the composites prepared using unmodified TiO_2_ NPs, the first and second DTG peaks were practically overlapped due to the close decomposition temperatures of HS and SS. On the other side, DTG peaks of PUC_M_ were nicely separated and distinct. Clearly, the incorporation of TiO_2_–LG NPs in the PU matrix was responsible for this, indicating that applied nano-filler is mostly embedded in the SS of PU, leading to the delay of thermal degradation of PCL. Additionally, in the DTG curves of each sample, there was one shoulder on the right side of the second DTG peak (*T*_sh2_, Table 4), which corresponds to the PCL decomposition. The enhancement of thermal stability was also detected after the incorporation of TiO_2_–LG NPs in poly(methyl methacrylate) [34] and polystyrene [35]. Furthermore, Stroea et al. demonstrated that the incorporation of silane-modified TiO_2_ NPs in PU based on PCL and poly(ethylene glycol) improves its thermal stability at a higher decomposition temperature range [39]. A similar result was obtained by Guo et al. after the addition of TiO_2_ NPs in waterborne polyurethane coatings [40].

### 3.6. Estimation of the Apparent Activation Energy of Thermal Degradation

**Figure 7:**
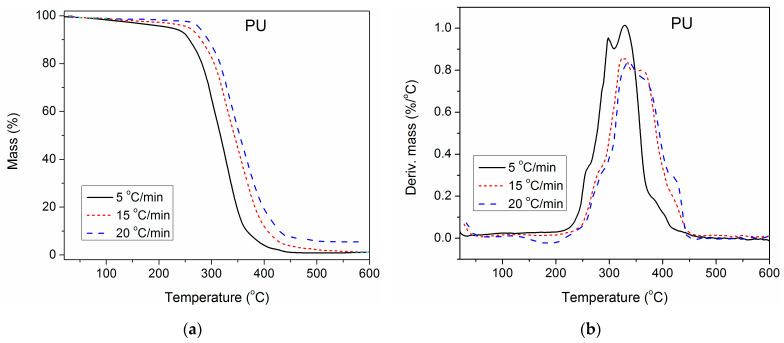
(**a**) TG and (**b**) DTG curves of pure PU, determined at different heating rates.

**Figure 8:**
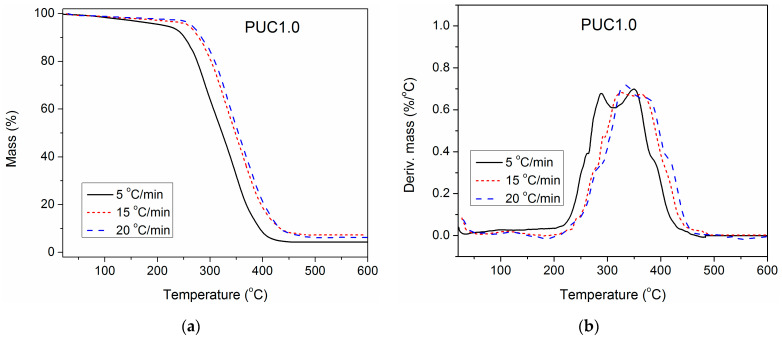
(**a**) TG and (**b**) DTG curves of PUC1.0, determined at different heating rates.

**Figure 9:**
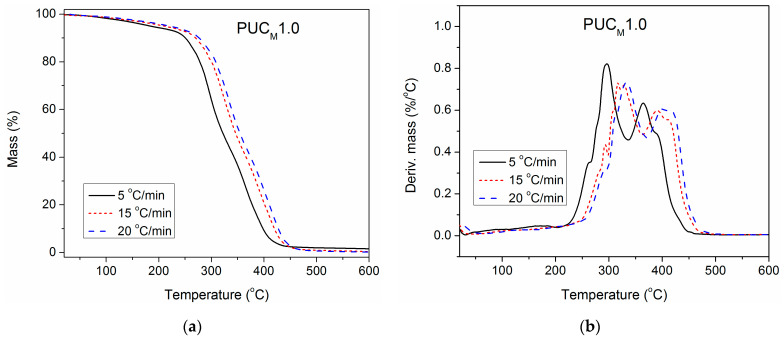
(**a**) TG and (**b**) DTG curves of PUC_M_1.0, determined at different heating rates.

To gain deeper insight into the impact of unmodified and surface-modified TiO_2_ NPs on the thermal stability of the synthesized PU, TGA of pure PU and composites containing 1.0 wt.% of unmodified and modified TiO_2_ NP was also performed at three additional heating rates (5, 15 and 20 °C/min), besides 10 °C/min. The obtained results are presented in Figure 7, Figure 8 and Figure 9. With the increase in heating rate, TG and DTG curves of all three examined samples shifted to higher temperatures, because of the lag in heat transfer when the heating rate was raised [41,42]. For complete thermal degradation, it is necessary for the examined samples to spend enough time at a specific degradation temperature. Therefore, at a higher heating rate, the time is reduced, and the measured TG and DTG curves move to the right. As a result, the first and second DTG peaks of pure PU are better separated at a heating rate of 5 °C/min, while at 20 °C/min, they are completely overlapped.

Thermal degradation rate of polymers can be written as [43,44,45]:

(1) $$\frac{d\alpha }{dt}=k\left(T\right)f(\alpha ),$$

where *α* represents the degree of conversion, which can be calculated using TGA results, i.e., values of the mass of the sample before thermal degradation (*w*_0_), mass at time *t* (*w_t_*) and mass at the end of degradation (*w*_f_) [45]:

(2) $$\alpha =\frac{w_{0}-w_{t}}{w_{0}-w_{f}},$$

Furthermore, in Equation (1), *f*(*α*) defines the model (mechanism) of the degradation reaction and *k*(*T*) is the constant of the degradation reaction rate, expressed by the Arrhenius relation [41]:

(3) $$k\left(T\right)=Ae^{\left(-\frac{E_{a}}{RT}\right)},$$

where *T* is absolute temperature, *A* is *the* pre-exponential factor, *E*_a_ represents the apparent activation energy of thermal degradation and *R* represents the universal gas constant (8.314 J/molK). By combining the Equations (1) and (3) and knowing that constant heating rate (*β* = d*T*/d*t*) is applied during the measurements, the following equation can be written:

(4) $$\beta \frac{d\alpha }{dT}=Af(\alpha )e^{\left(-\frac{E_{a}}{RT}\right)}.$$

Due to the complexity of the thermal degradation mechanism of polymers and the fact that their degradation rarely occurs as a single process, model-free isoconversional methods are often applied to examine its multiplicity. By using model-independent methods, it is not required to know the form of *f*(*α*), since these methods assume that at constant *α*, only temperature influences the rate of the degradation reaction (*f*(*α*) = const.) [46]. In this manner, it is possible to calculate *E*_a_ at different degrees of conversion. One of the most common integral isoconversional methods is the Ozawa–Flynn–Wall (OFW) method, described by the following equation [47,48]:

(5) $$ln\beta =ln\frac{{AE}_{a}}{Rg(\alpha )}-5.3305-1.052\frac{E_{a}}{RT},$$

where *g*(*α*) is an integral conversion function. From the slope of the linear dependence ln*β* vs. 1/*T*, values of the *E*_a_ of the investigated samples for *α* = const. can be calculated. The Kissinger–Akahira–Sunose (KAS) method is also a frequently used integral method in the form of [49,50]:

(6) $$ln\frac{\beta }{T^{2}}=ln\frac{AR}{E_{a}g(\alpha )}-\frac{E_{a}}{RT}.$$

**Figure 10:**
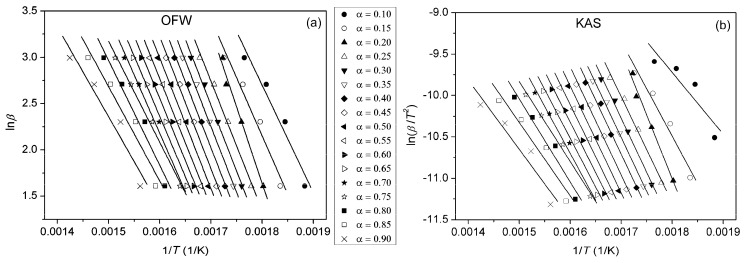
Plots obtained using (**a**) OFW and (**b**) KAS methods for pure PU, determined at different *α*.

**Figure 11:**
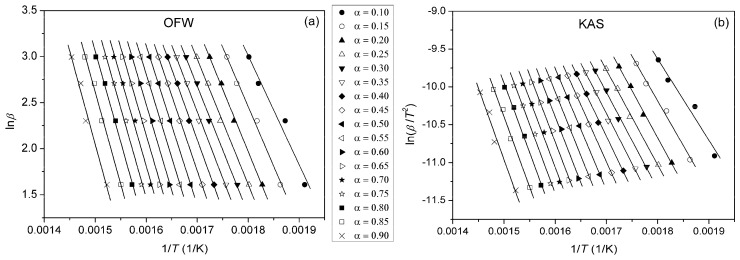
Plots obtained using (**a**) OFW and (**b**) KAS methods for PUC1.0, determined at different *α*.

**Figure 12:**
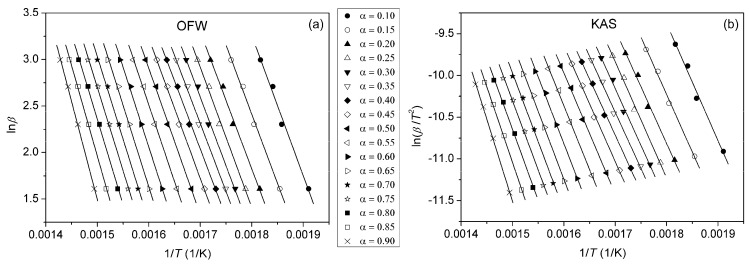
Plots obtained using (**a**) OFW and (**b**) KAS methods for PUC_M_1.0, determined at different *α*.

By plotting ln(*β*/*T*^2^) vs. 1/*T* at different heating rates, *E*_a_ can be calculated from the slope. Plots obtained using OFW and KAS methods for pure PU, PUC1.0 and PUC_M_1.0 at different *α* are given in Figure 10, Figure 11 and Figure 12. The third model-free isoconversional method used here is a differential Friedman method [51]:

(7) $$ln\left(\beta \frac{d\alpha }{dT}\right)=lnA+lnf(\alpha )-\frac{E_{a}}{RT}.$$

**Figure 13:**
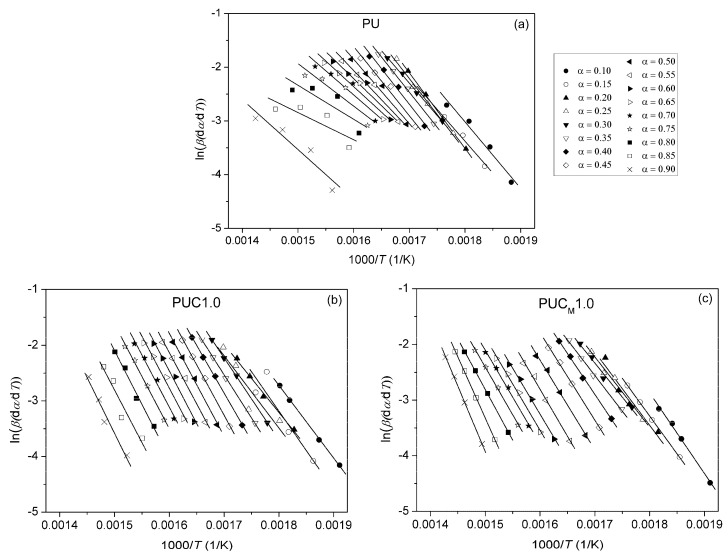
Plots obtained using Friedman method for (**a**) pure PU, (**b**) PUC1.0 and (**c**) PUC_M_1.0, determined at different *α*.

In this case, *E*_a_ can be calculated from the slope of linear plots ln[*β*(d*α*/d*T*)] vs. 1/*T* at different heating rates. Plots obtained by applying the Friedman method are presented in Figure 13.

For the pure PU, lines fitted using all three free-model methods at low (0.1 < *α* < 0.2) and high (0.8 < *α* < 0.9) conversion degrees (Figure 10 and Figure 13) had relatively low linear correlation coefficients (R^2^ < 0.98) and large deviations in parallelism, probably due to the experimental error induced by the presence of noise in the obtained data. On the other hand, the reliability of the model-free isoconversional methods used for PUC1.0 and PUC_M_1.0 was ensured by the strong linear relationship observed over a wide temperature range and high value of linear correlation coefficients (R^2^ > 0.99). The difference between integral (OFW, KAS) and differential (Friedman) methods was clearly visible from the results presented in Figure 10, Figure 11, Figure 12 and Figure 13. The wavy shape of the plots in Figure 13 was a consequence of the differentiation of integral TG data. Contrarily, linear plots fitted according to the OFW and KAS methods were gathered in a sequence of parallel lines, with a slight but not negligible change in the slope in the range of *α* < 0.5. For each examined sample, the slope change appeared at different values of *α* and this was more visible from the data obtained using the Friedman method. These results confirm that the thermal degradation of pure PU and synthesized composites cannot be described as a one-step process, but most likely decomposition occurs through two steps with different mechanisms of thermal decomposition. The low visibility of slope displacement is probably a result of partial overlapping of the first and second decomposition steps.

**Figure 14:**
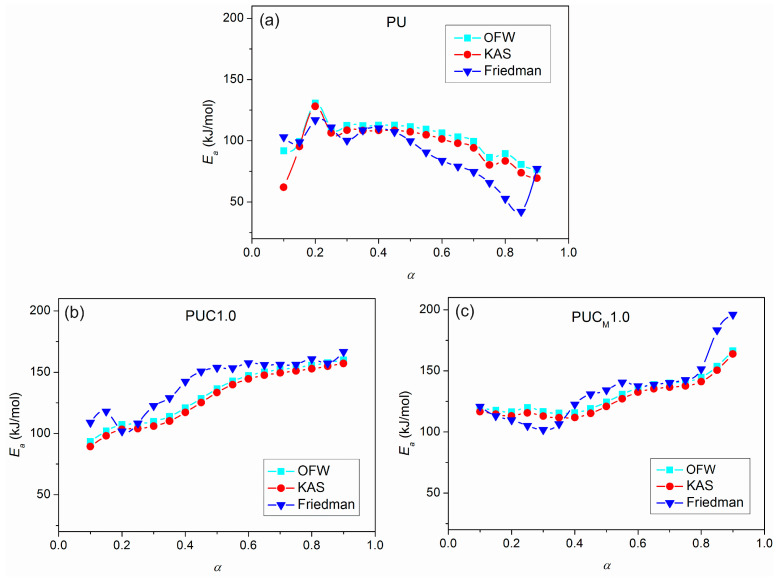
Dependence of *E*_a_ vs. *α* for (**a**) pure PU, (**b**) PUC1.0 and (**c**) PUC_M_1.0, calculated according to OFW, KAS and Friedman methods.

From the determined values of the slopes of the straight lines obtained after fitting TG results according to the applied free-model methods, the dependences of *E*_a_ on conversion for pure PU and composites were estimated and are shown in Figure 14. It can be observed that the deviations in the parallelism of OFW, KAS and Friedman plots for pure PU at lower and higher conversions (Figure 10 and Figure 13) are clearly reflected in the values of *E*_a_. The derived dependences are in line with previously made observations considering the complexity of thermal decomposition of the examined samples, indicating that it is more accurate to check the complexity of certain reactions from the *E*_a_ = *f*(*α*) dependence. Namely, only when *E*_a_ is constant at all conversion degrees, can it be concluded that thermal degradation occurred as a simple, one-step process. However, that was not the case with the samples investigated here. The profiles of the *E*_a_ = *f*(*α*) dependences evaluated using the OFW and KAS methods were quite similar, with slightly higher values of *E*_a_(OFW). The change in *E*_a_ with conversion calculated according to the Friedman method had a similar trend, but somewhat different profile for all three samples, due to the different approaches (differential and integral). Furthermore, both the profile and trend of *E*_a_ = *f*(*α*) plots for pure PU and the prepared composites differed from each other. The apparent activation energy of PU decreased with conversion, while the opposite was obtained for the composites, most likely due to the different mechanism of thermal decomposition during the second step. According to Vyazovkin, the shape of the *E*_a_ = *f*(*α*) dependences can give certain, oversimplified indications about the degradation mechanism involved in the process [52,53]. A decrease in *E*_a_ with conversion indicates cleavage of bonds with smaller energies and diffusion of small molecules. On the other hand, a increase in *E*_a_ with conversion is associated with the appearance of parallel degradation reactions, and the one with the higher *E*_a_ has a larger contribution to the final rate of the process. In the case of PUC and PUC_M_, these results clearly confirm the positive impact of unmodified and surface-modified TiO_2_ NPs on the thermal stability and aging resistance of PU.

A detailed insight into TG data, results gathered after applying free-model methods, and *E*_a_ = *f*(*α*) dependences allowed the estimation of the most probable conversion ranges of each decomposition step (*α*′ and *α*″), and evaluation of the corresponding average *E*_a_ for all investigated samples (Table 5). The beginning of thermal degradation of PCL (the second step) moves to higher conversions in the following order: PU < PUC1.0 < PUC_M_1.0. This is a result of the effect of NPs on the thermal stability of PU. Also, higher *E*_a_ values of the composites during the second step of decomposition support the dominant role of incorporated TiO_2_ NPs in influencing the thermal performance of PU (Table 5). As expected, the average *E*_a_ of PUC_M_1.0 in the second step of thermal degradation was the highest, according to all three applied model-free methods. All these speak in favor of the beneficial impact of embedding TiO_2_ NPs, especially those surface modified with lauryl gallate, on the thermal stability of the PU matrix.

## 4. Conclusions

Polyurethane composites were fabricated by embedding different portions of unmodified and surface-modified TiO_2_ NPs in an environmentally safe PU network based on PCL. Surface modification of TiO_2_ NPs was done with lauryl gallate in order to facilitate interfacial interaction of hydrophobic PU with hydrophilic TiO_2_ NPs. With each type of TiO_2_ NPs (unmodified and surface-modified), three samples containing 0.5, 1.0 and 2.0 wt.% of TiO_2_ NPs were prepared.

Results obtained by FTIR spectroscopy confirmed the structure of the prepared pure PU and the composites, as well as the surface modification of TiO_2_ NPs. Swelling measurements of the prepared samples in THF revealed that the addition of unmodified and surface-modified TiO_2_ NPs, regardless of their content, increases the swelling ability of the PU network, i.e., reduces its crosslinking density. SEM results disclosed the presence of TiO_2_ agglomerates and indicated that surface-modified TiO_2_ NPs are better dispersed in the polymer matrix than unmodified ones. According to the tensile tests, the mechanical properties of the PU matrix deteriorate after the inclusion of unmodified or surface-modified TiO_2_ NPs, which also indicates the presence of agglomerates.

From the TG and DTG curves, it was concluded that the thermal decomposition of pure PU and the prepared composites occurred as a two-step process. The positive influence of TiO_2_ NPs on the thermal stability of PU was detected through the shift in the second DTG maximum to the higher temperatures in the DTG curves of composites, and this effect is more pronounced in composites prepared using TiO_2_ NPs surface modified with lauryl gallate. The aliphatic part of lauryl gallate is obviously long enough to secure better dispersion of TiO_2_–LG NPs in PU, simultaneously leading to the improvement of the PU thermal properties. It was also concluded that TiO_2_–LG NPs are mostly embedded in the SS of PU, leading to delayed thermal degradation of PCL. The estimation of the *E*_a_ = *f*(*α*) showed that thermal degradation of all the samples occurs through two steps with different mechanisms of thermal decomposition. For pure PU, it was estimated that *E*_a_ decreases with conversion, indicating the cleavage of bonds with smaller energies and the diffusion of small molecules. On the other hand, *E*_a_ of composites increased with conversion, which is associated with the appearance of parallel degradation reactions, where the reaction with a higher *E*_a_ has a larger contribution to the final rate of the process. These results clearly confirm the dominant role of incorporated TiO_2_ NPs on the positive course of thermal stability and aging resistance of PUs.

## Figures and Tables

**Table 1:** Results obtained by Gaussian deconvolution on C=O stretching region of prepared samples.

Sample	Area (%)				
	C=O _free urethane_ 1729 cm^−1^	C=O _free ester_ 1722 cm^−1^	C=O _disorder_ _H-bonded urethane_ 1705 cm^−1^	C=O _order_ _H-bonded urethane_ 1695 cm^−1^	C=O _H-bonded ester_ 1640 cm^−1^
PU	40.4	23.6	8.5	15.6	11.9
PUC0.5	36.9	11.2	4.6	31.2	16.1
PUC1.0	36.7	11.3	4.2	30.8	17.0
PUC2.0	33.2	12.5	5.7	29.8	18.8
PUC_M_0.5	15.4	19.6	20.1	35.2	9.7
PUC_M_1.0	9.9	27.8	15.9	33.8	12.6
PUC_M_2.0	6.7	36.5	14.9	28.7	13.2

**Table 2:** Values of density (*ρ*), volume fraction of the crosslinked polymer in the swollen specimen (*V*), crosslinking density (*ν*), average molecular weight of polymer chain between crosslinks (*M*_c_) and gel content (gel%) calculated from swelling measurements in THF of the prepared samples.

Sample	*ρ* (g cm^−3^)	*V*	*ν* × 10^4^ (mol cm^−3^)	*M*_c_ (g mol^−1^)	Gel% (wt.%)
PU	1.011 ± 0.010	0.320 ± 0.025	6.25 ± 0.29	1620 ± 170	79.0 ± 0.2
PUC0.5	1.017 ± 0.016	0.233 ± 0.012	2.93 ± 0.31	3470 ± 453	70.0 ± 0.1
PUC1.0	1.024 ± 0.015	0.244 ± 0.031	3.38 ± 0.42	3030 ± 391	67.1 ± 0.1
PUC2.0	1.031 ± 0.017	0.298 ± 0.020	5.66 ± 0.45	1820 ± 211	77.1 ± 0.2
PUC_M_0.5	1.022 ± 0.016	0.213 ± 0.009	2.45 ± 0.37	4160 ± 550	62.2 ± 0.1
PUC_M_1.0	1.030 ±0.018	0.259 ± 0.028	3.99 ± 0.32	2580 ± 198	74.5 ± 0.2
PUC_M_2.0	1.037 ± 0.020	0.265 ± 0.011	4.31 ± 0.45	2400 ± 215	74.6 ± 0.1

**Table 3:** Tensile strength, elongation at break and Young’s modulus of prepared samples.

Sample	Tensile Strength (MPa)	Elongation at Break (%)	Young’s Modulus (MPa)
PU	14.2 ± 1.2	8.2 ± 0.6	678.2 ± 37.1
PUC0.5	13.8 ± 1.0	5.4 ± 0.4	522.9 ± 29.5
PUC1.0	17.1 ± 1.5	10.8 ± 0.7	663.4 ± 33.4
PUC2.0	4.2 ± 0.3	0.6 ± 0.1	529.9 ± 28.1
PUC_M_0.5	10.9 ± 0.8	3.0 ± 0.2	574.9 ± 30.0
PUC_M_1.0	11.6 ± 0.9	5.1 ± 0.3	563.3 ± 26.8
PUC_M_2.0	12.5 ± 1.1	6.3 ± 0.5	597.0 ± 31.3

**Table 4:** Characteristic temperatures of thermal degradation *T*_10_ and *T*_50_ (at 10 and 50% weight loss, respectively), temperatures of two shoulders (*T*_sh1_ and *T*_sh2_) and temperatures of maximum thermal degradation rate (*T*_max1_ and *T*_max2_) of the pure PU and prepared composites, at a heating rate of 10 °C/min.

Sample	*T*_10_ (°C)	*T*_50_ (°C)	*T*_sh1_ (°C)	*T*_max1_ (°C)	*T*_max2_ (°C)	*T*_sh2_ (°C)
PU	269	332	270	323	347	401
PUC0.5	265	335	274	316	352	403
PUC1.0	262	335	270	318	351	400
PUC2.0	263	335	279	318	352	400
PUC_M_0.5	268	339	278	317	373	402
PUC_M_1.0	269	344	277	319	382	405
PUC_M_2.0	258	344	277	319	383	405

**Table 5:** Average apparent activation energy (*E*_a_) and average correlation coefficient (R^2^) for first (*α*′) and second (*α*″) step of thermal degradation of examined samples, evaluated using OFW, KAS and Friedman model-free methods.

*α*	*E*_a_ (OFW) (kJ/mol)	R^2^ (OFW)	*E*_a_ (KAS) (kJ/mol)	R^2^ (KAS)	*E*_a_ (Friedman) (kJ/mol)	R^2^ (Friedman)
PU						
0.10 < *α*′ < 0.25	108 ± 15	0.9621	98 ± 23	0.9521	107 ± 7	0.9797
0.30 < *α*″ < 0.90	101 ± 13	0.9936	96 ± 14	0.9841	84 ± 21	0.9298
PUC1.0						
0.10 < *α*′ < 0.30	104 ± 6	0.9889	100 ± 6	0.9846	112 ± 7	0.9516
0.35 < *α*″ < 0.90	139 ± 14	0.9957	136 ± 15	0.9966	149 ± 9	0.9548
PUC_M_1.0						
0.10 < *α*′ < 0.40	117 ± 1	0.9955	114 ± 2	0.9932	111 ± 7	0.9831
0.45 < *α*″ < 0.90	143 ± 13	0.9973	141 ± 13	0.9964	153 ± 21	0.9957

## Data Availability

The raw data supporting the conclusions of this article will be made available by the authors on reasonable request.
